# Advanced materials nanocharacterization

**DOI:** 10.1186/1556-276X-6-107

**Published:** 2011-01-31

**Authors:** Filippo Giannazzo, Pierre Eyben, Jacek Baranowski, Jean Camassel, Stefan Lányi

**Affiliations:** 1 CNR-IMM, Strada VIII, 5, 95121 Catania, Italy; 2 Imec Kapeldreft, 75 3001 Leuven, Belgium; 3 Institute of experimental Physics, ul. Hoża 69 Warsaw 00-681, Poland; 4 Groupe d'Etude des Semiconducteurs, Université Montpellier 2, 34095 Montpellier Cedex 5, France; 5 Institute of Physics, SAS Dúbravská cesta 9 845 11 Bratislava, Slovakia

## Abstract

This special issue of *Nanoscale Research Letters *contains scientific contributions presented at the Symposium D *"Multidimensional Electrical and Chemical Characterization at the Nanometer-scale of Organic and Inorganic Semiconductors" *of the E-MRS Fall Meeting 2010, which was held in Warsaw, Poland from 13^th ^to 17^th ^September, 2010.

## Editorial

This special issue of Nanoscale Research Letters contains scientific contributions presented at the Symposium D *"Multidimensional Electrical and Chemical Characterization at the Nanometer-scale of Organic and Inorganic Semiconductors" *of the E-MRS Fall Meeting 2010, which was held in Warsaw, Poland from 13^th ^to 17^th ^September, 2010.

The Symposium, attended by more than 100 participants from different countries and regions, was aimed at bringing together scientists and engineers working on various aspects of semiconductor research in both academic and industrial environments to discuss the metrology aspects and present innovative solutions. Hot topics covered at the symposium were:

• Advanced scanning probe microscopy (AFM, SCM, SSRM, SKFM, SNOM)

• Advanced electron microscopy (HRTEM, EFTEM,electron-holography,...)

• Optical characterization (μRaman, photo- electro- luminescence)

• 2D and 3D chemical mapping of materials at the nanometer scale

• Metrology in advanced semiconductors (Ge, SiGe, InGaAs, SiC, GaN, AlGaN..), semiconductor heterointerfaces and semiconductor nanowires.

• Characterization of organic materials and carbon- based materials (nanotubes, graphene).

• Local measurements (morphological, electronic and transport properties) in graphene with high spatial resolution

This special issue is a peer-reviewed collection of 25 papers covering most of the scientific issues addressed during the symposium.

We would like to express our appreciation to all members of the Scientific Committee for their invaluable suggestions and selection of the invited speakers and scientific contributions.

We also thank the authors for their excellent contributions, as well as the referees whose feedback and comments ensured the high quality of this special issue.

EMRS Conference Chairmen:

- Andrzej MYCIELSKI, Institute of Physics Polish Academy of Sciences, Warsaw, Poland.

- Francesco PRIOLO, University of Catania, Dipartimento di Fisica e Astronomia,Catania, Italy.

- Urszula NARKIEWICZ, Faculty of Chemical Engineering, West Pomeranian University of Technology, Poland.

- George SMITH, Department of Materials Oxford University, United Kingdom.

Symposium D Chairmen:

- Filippo Giannazzo, CNR-IMM, Strada VIII, 5, 95121 Catania, Italy

- Pierre Eyben, Imec Kapeldreft, 75 3001 Leuven, Belgium

- Jacek Baranowski, Institute of experimental Physics, ul. Hoża 69 Warsaw 00-681, Poland

- Jean Camassel, Groupe d'Etude des Semiconducteurs, Université Montpellier 2, 34095 Montpellier Cedex 5, France

- Stefan Lányi, Institute of Physics, SAS Dúbravská cesta 9 845 11 Bratislava, Slovakia

Guest Editors:

- Filippo Giannazzo, CNR-IMM, Strada VIII, 5, 95121 Catania, Italy

- Pierre Eyben, Imec Kapeldreft, 75 3001 Leuven, Belgium

- Jacek Baranowski, Institute of experimental Physics, ul. Hoża 69 Warsaw 00-681, Poland

- Jean Camassel, Groupe d'Etude des Semiconducteurs, Université Montpellier 2, 34095 Montpellier Cedex 5, France

- Stefan Lányi, Institute of Physics, SAS Dúbravská cesta 9 845 11 Bratislava, Slovakia

